# Supplementary material for: Mapping aquifer salinity gradients and effects of oil field produced water disposal using geophysical logs: Elk Hills, Buena Vista and Coles Levee Oil Fields, San Joaquin Valley, California
Source: PLoS One. 2022 Mar 28;17(3):e0263477. doi: 10.1371/journal.pone.0263477 (PMC8959183; doi:10.1371/journal.pone.0263477)
Supplement: S1 File — (DOCX) [file pone.0263477.s001.docx]

**S1 File**

**Method for generating the Water Surface Elevation (WSE) maps**

The WSE values, here written as vector $\boldsymbol{u}$, are modeledd with a distribution

𝒖 | 𝝁 ~ 𝒩(𝝁,Σ), (1)

where the trend 𝝁 is derived from the space-time location of each WSE measurement. Specifically,

$\boldsymbol{\mu}=F^{T}\boldsymbol{w}$, (2)

where 𝒘 is a vector of parameters and 𝐹 is a 10×𝑁 matrix where each column 𝑖 is a featurization of the space-time location (𝑥_𝑖_, 𝑦_𝑖_, 𝑡_𝑖_) of observation 𝑖. To construct F, first create a featurization matrix $A$, also of dimensions 10×𝑁, where each column contains polynomial terms up to two degrees, i.e. column $\boldsymbol{a}_{i}=\left( 1, x_{i},y_{i},t_{i},x_{i}^{2},y_{i}^{2},t_{i}^{2}, x_{i}y_{i}, x_{i}t_{i}, y_{i}t_{i} \right)^{T}$. Each row of $A$ besides the first is then translated and scaled so that their means and variances are 0 and 1, yielding $F$.. To avoid overfitting, 𝒘 is assigned a diffuse prior and is marginalized over:

𝒘 ~ 𝒩(0, 𝛼𝐼), (3)

where 𝐼 is an identity matrix and 𝛼 was set to the square of the sample range of WSE so as to be large enough to permit reasonable trend coefficient values.

In Eq. 1, $\Sigma$ is a covariance matrix based on three stacked gamma-exponential covariance functions. Thus $\Sigma=\Sigma^{\left( 1 \right)}+\Sigma^{\left( 2 \right)}+\Sigma^{\left( 3 \right)}$, where entry $\sigma_{i,j}^{\left( k \right)}$ of component $\Sigma^{\left( k \right)}$ is

$\sigma_{i,j}^{\left( k \right)}=s^{\left( k \right)}\exp\left[ -\left( \frac{d^{\left( k \right)}\left( i,j \right)}{r^{\left( k \right)}} \right)^{\gamma^{\left( k \right)}} \right]+n^{\left( k \right)}\delta^{\left( k \right)}\left( i,j \right).$ (4)

The parameters are (to use terms from kriging) sill $s^{\left( k \right)}$, range $r^{\left( k \right)}$, shape $\gamma^{\left( k \right)}$, and nugget $n^{\left( k \right)}$. A shape parameter has bounds $0<\gamma^{\left( k \right)}\leq2$, range $r^{\left( k \right)}$ must be positive, and the other parameters must be non-negative. Each covariance component uses a different distance function $d^{\left( k \right)}\left( i,j \right)$. The Dirac delta function $\delta^{\left( k \right)}\left( i,j \right)$ returns 1 if $d^{\left( k \right)}\left( i,j \right)=0$, else it returns 0.

The first distance function $d^{\left( 1 \right)}\left( i,j \right)$ is time-only (space-invariant): it returns the time elapsed between measurements $i$ and $j$. This covariance component models regional rain patterns that would affect WSE in the entire area of study. The nugget for this component $n^{\left( 1 \right)}$ is unused and is set to zero.

The second distance function $d^{\left( 2 \right)}\left( i,j \right)$ is time-invariant (space-only): it returns the two-dimensional Euclidean distance between measurements $i$ and $j$. This covariance component models time-invariant phenomena such as longstanding waste disposal practices and the effect of ground surface elevation on WSE. The nugget parameter for this component $n^{\left( 2 \right)}$ models the effect of well identity on WSE.

The third distance function $d^{\left( 3 \right)}\left( i,j \right)$ returns the Euclidean distance between measurements $i$ and $j$ in space and time, after time has been scaled by an anisotropy parameter $\kappa$. The nugget parameter for this component $n^{\left( 3 \right)}$ models both measurement error, and excursions in WSE at the time and place of individual measurements. Since we cannot know how much of the nugget to attribute to either, we conservatively attribute all of it to the latter (unpredictability in actual WSE) when plotting WSE prediction error.

In sum, there are twelve scalar geostatistical parameters in the construction of the covariance matrix: $s^{\left( 1 \right)},r^{\left( 1 \right)},\gamma^{\left( 1 \right)},s^{\left( 2 \right)},r^{\left( 2 \right)},\gamma^{\left( 2 \right)},n^{\left( 2 \right)},s^{\left( 3 \right)},r^{\left( 3 \right)},\gamma^{\left( 3 \right)},n^{\left( 3 \right)},\kappa$.

Combining Equations 1 and 2 by marginalizing over 𝒘, we obtain

$\boldsymbol{u}\mathcal{\sim N}\left( 0, \alpha F^{T}F+\Sigma\right)$. (5)

This equation yields an expression for model likelihood in terms of the model’s twelve geostatistical parameters. The likelihood function was written in TensorFlow [66] and maximized using the Adam optimizer [67]. Fitted parameters are shown in S2 Table and maps showing the probability distribution for WSE at each location is shown in S5 Fig. The model code, input, and output data are available from Stephens et al. [23].
