# Supplementary figures and images for: Mapping aquifer salinity gradients and effects of oil field produced water disposal using geophysical logs: Elk Hills, Buena Vista and Coles Levee Oil Fields, San Joaquin Valley, California
Source: PLoS One. 2022 Mar 28;17(3):e0263477. doi: 10.1371/journal.pone.0263477 (PMC8959183; doi:10.1371/journal.pone.0263477)

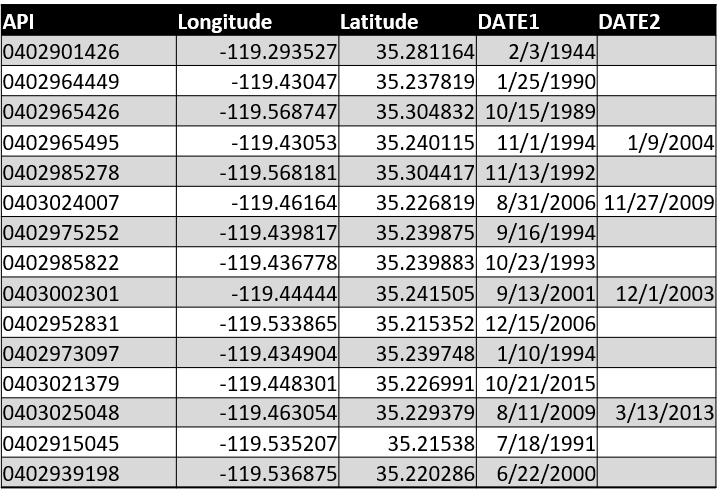

Supplement: S1 Table — Date 1 is the date of the first surface expression in the well and Date2 is the date of the second surface expression (if any). Data from well history files available from CalGEM’s online Well Files [51]. (TIF) [file pone.0263477.s002.tif]

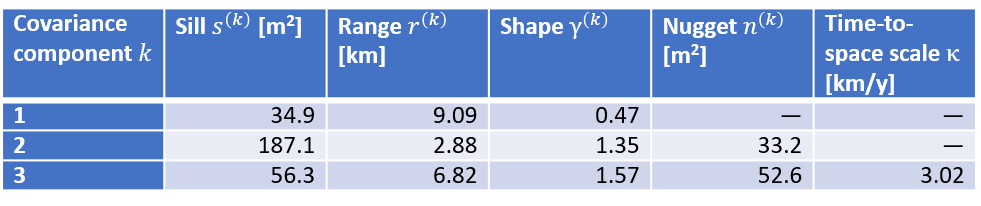

Supplement: S2 Table — (TIF) [file pone.0263477.s003.tif]

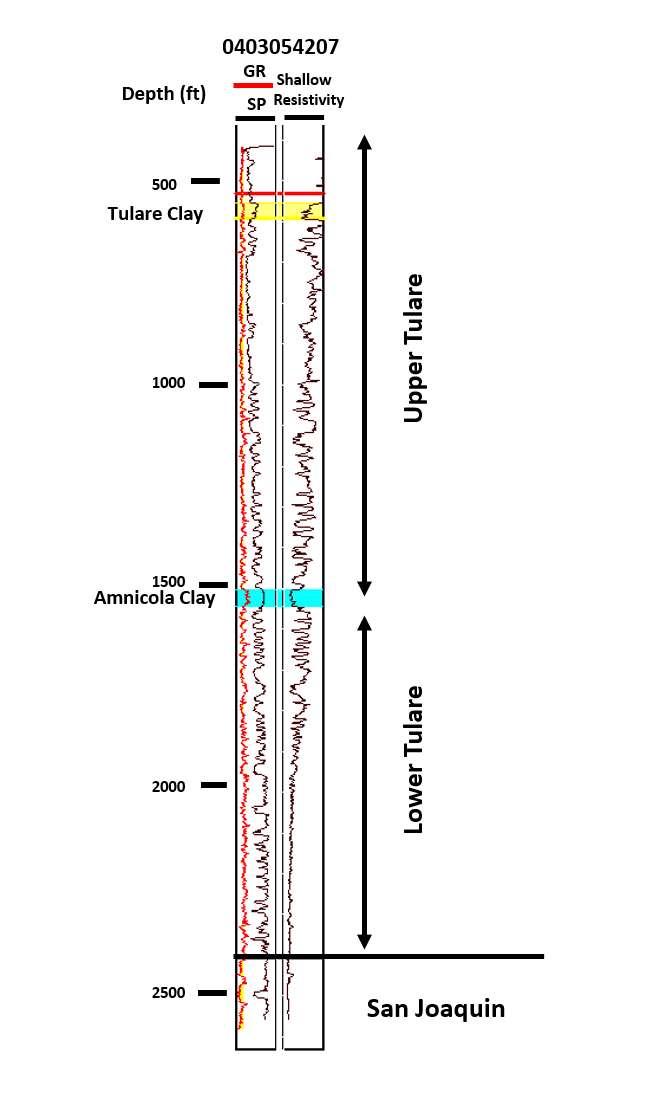

Supplement: S1 Fig — Left track contains GR = Gamma Ray log in red, SP = spontaneous potential log in black and right track contains shallow resistivity log in black. The yellow interval is the Tulare clay and the blue interval is the Amnicola clay. The Amnicola clay divides the upper and lower Tulare Formation for this study. Well location shown in Fig 3. (TIF) [file pone.0263477.s004.tif]

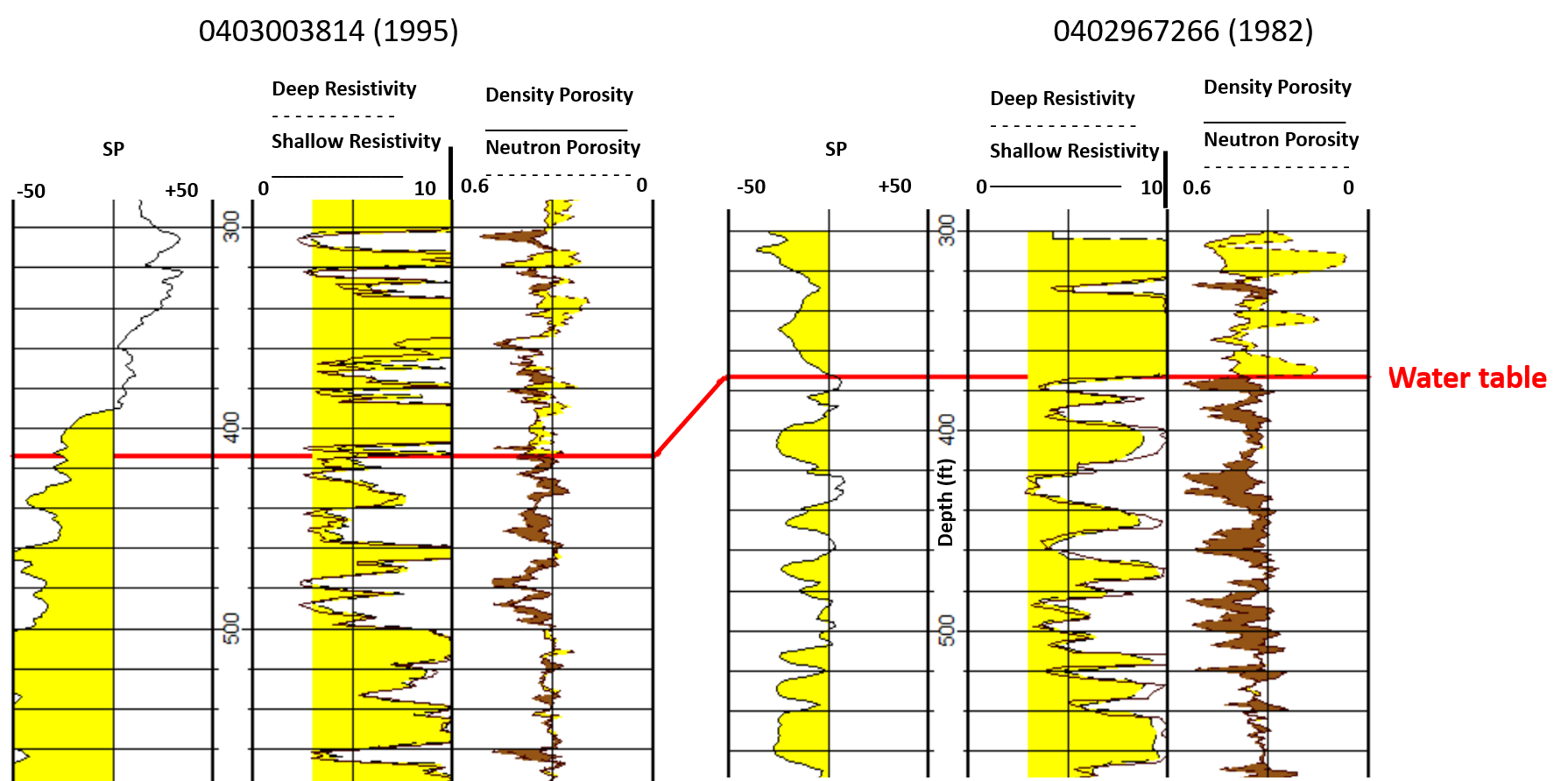

Supplement: S2 Fig — Negative SP is shaded yellow and resistivity curve is shaded yellow at values greater than three ohms. Resistivity readings are typically higher in unsaturated sands above the water table—particularly if the groundwater is brackish. Density-neutron log is shaded yellow in areas where the neutron porosity is lower than the density—a phenomenon known as cross-over that indicates the presence of air or natural gas. The top of the water table is picked at the base of cross-over in the density-neutron log. The density-neutron log is shaded brown in intervals where the neutron curve reads higher porosity than the density curve—the larger the difference, the greater the volume of clay within the formation. Clean sand intervals typically have little to no separation between the curves. (TIF) [file pone.0263477.s005.tif]

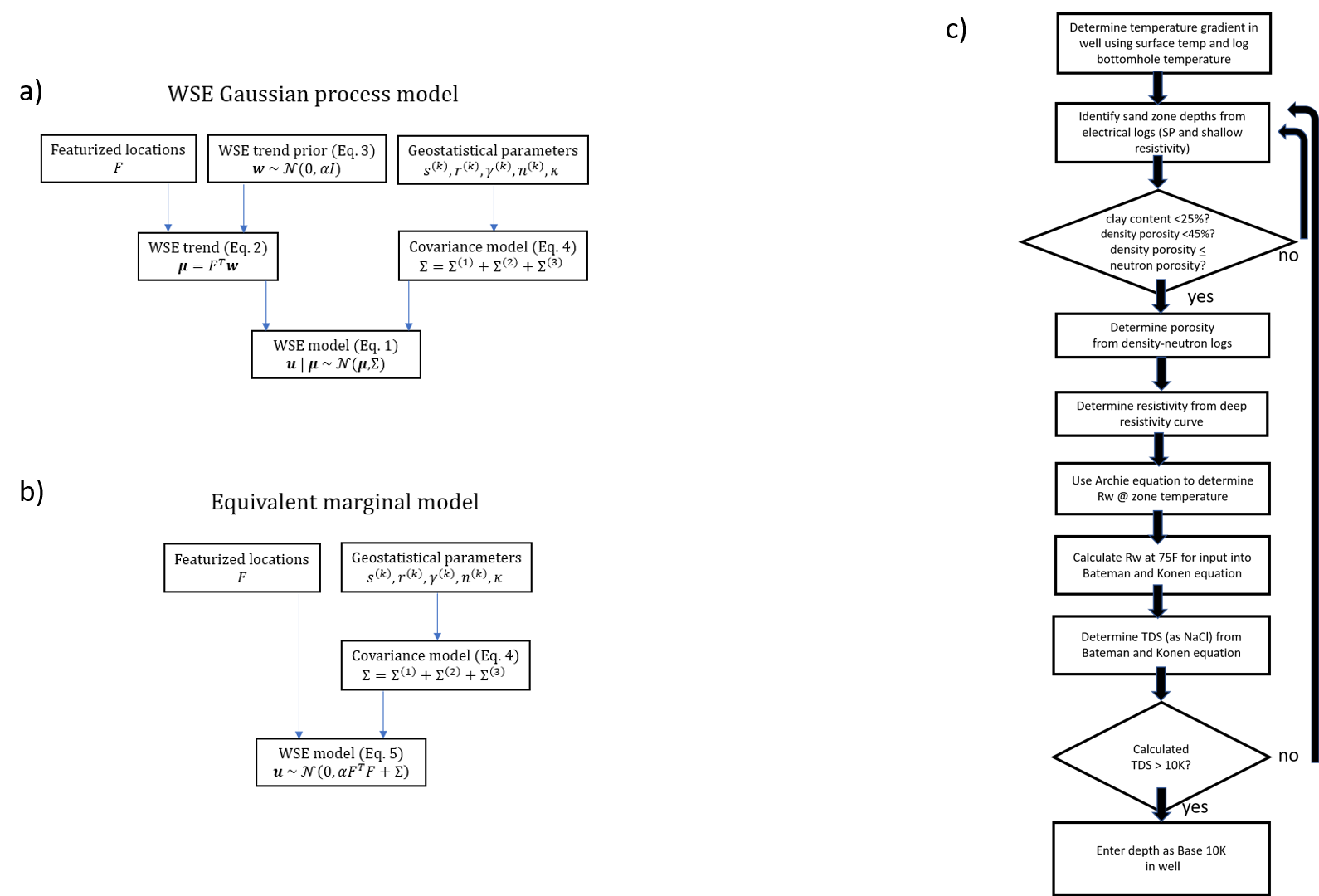

Supplement: S3 Fig — a) A probabilistic generative model of WSE data, as described in S1 File and b) an equivalent model, used for computing model likelihood. c) Flow chart showing steps used to calculate salinity from geophysical logs in Fig 8 and construction of the Base 10K map in Fig 9. (TIF) [file pone.0263477.s006.tif]

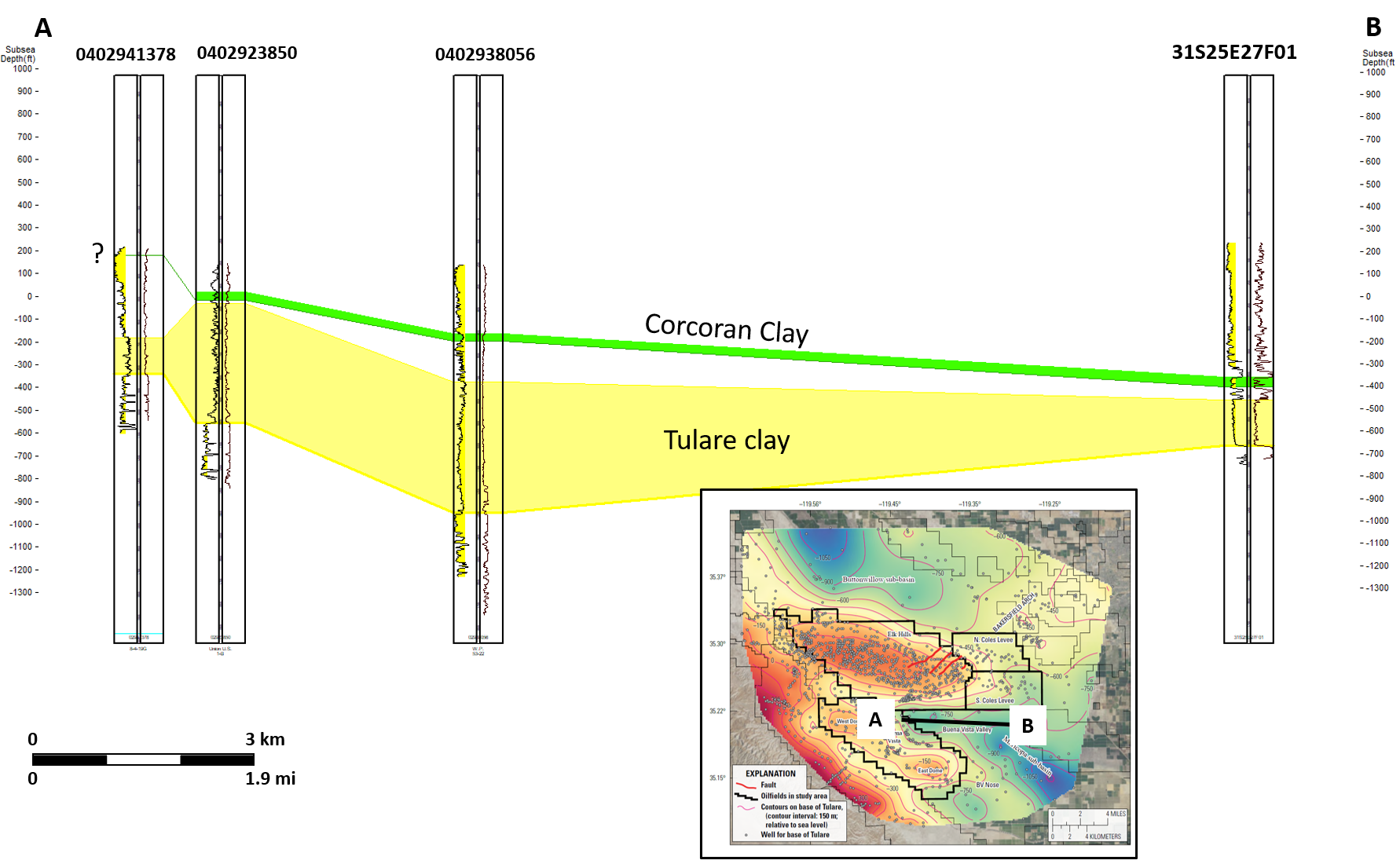

Supplement: S4 Fig — Corcoran Clay picks based on Croft’s [45] E-clay picks in wells 31S25E27F01 and 0402938056. Tulare clay picks from this study (note that SP curve is reversed in 31S25E27F01). Air photo base on inset map from https://basemap.nationalmap.gov/arcgis/rest/services/USGSImageryOnly/MapServer/. (TIF) [file pone.0263477.s007.tif]

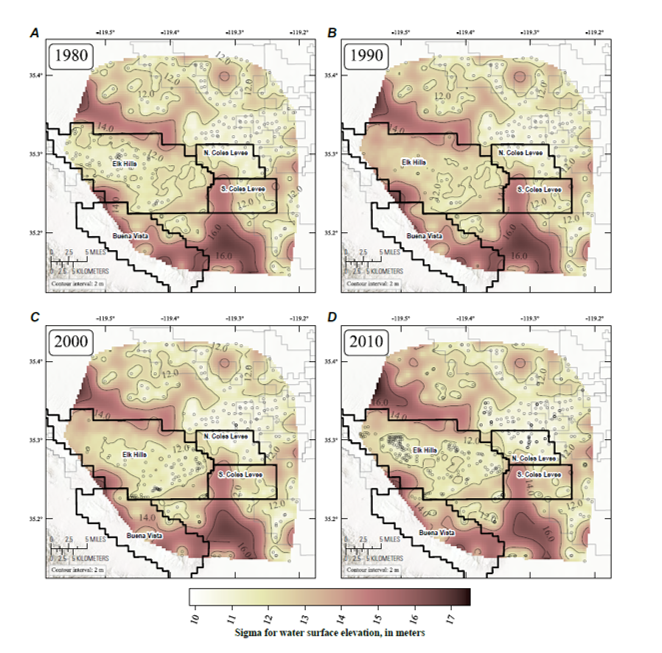

Supplement: S5 Fig — These maps provide a probability distribution for WSE at each location; therefore, we can quantify the uncertainty associated with each WSE prediction. At each location, the model provides a mean and variance, taking the square root of the variance gives the standard deviation (σ). The σ maps should be considered with the WSE maps (Fig 7) to understand which zones have higher or lower uncertainty. The uncertainty estimates are a function of distance from the input data. Uncertainty is lower when a WSE prediction is near a water table measurement (in space or time), conversely, predictions farther from input data have higher associated uncertainty. For example, on S5b Fig (1990) high uncertainties indicate input data are rather limited in the Elk Hills Oil Field which creates a data gap with the adjacent groundwater basin to the northeast. Therefore, caution should be used when interpreting the WSE predictions in the corresponding location on the WSE map (Fig 7B). By 2000 and 2010 more data are available in eastern Elk Hills and the uncertainty lowers. Basemap from Esri. "World Hillshade" [basemap]. Scale Not Given. "World Hillshade". July 9, 2015. https://www.arcgis.com/home/item.html?id=1b243539f4514b6ba35e7d995890db1d. (September 30, 2021). (TIF) [file pone.0263477.s008.tif]

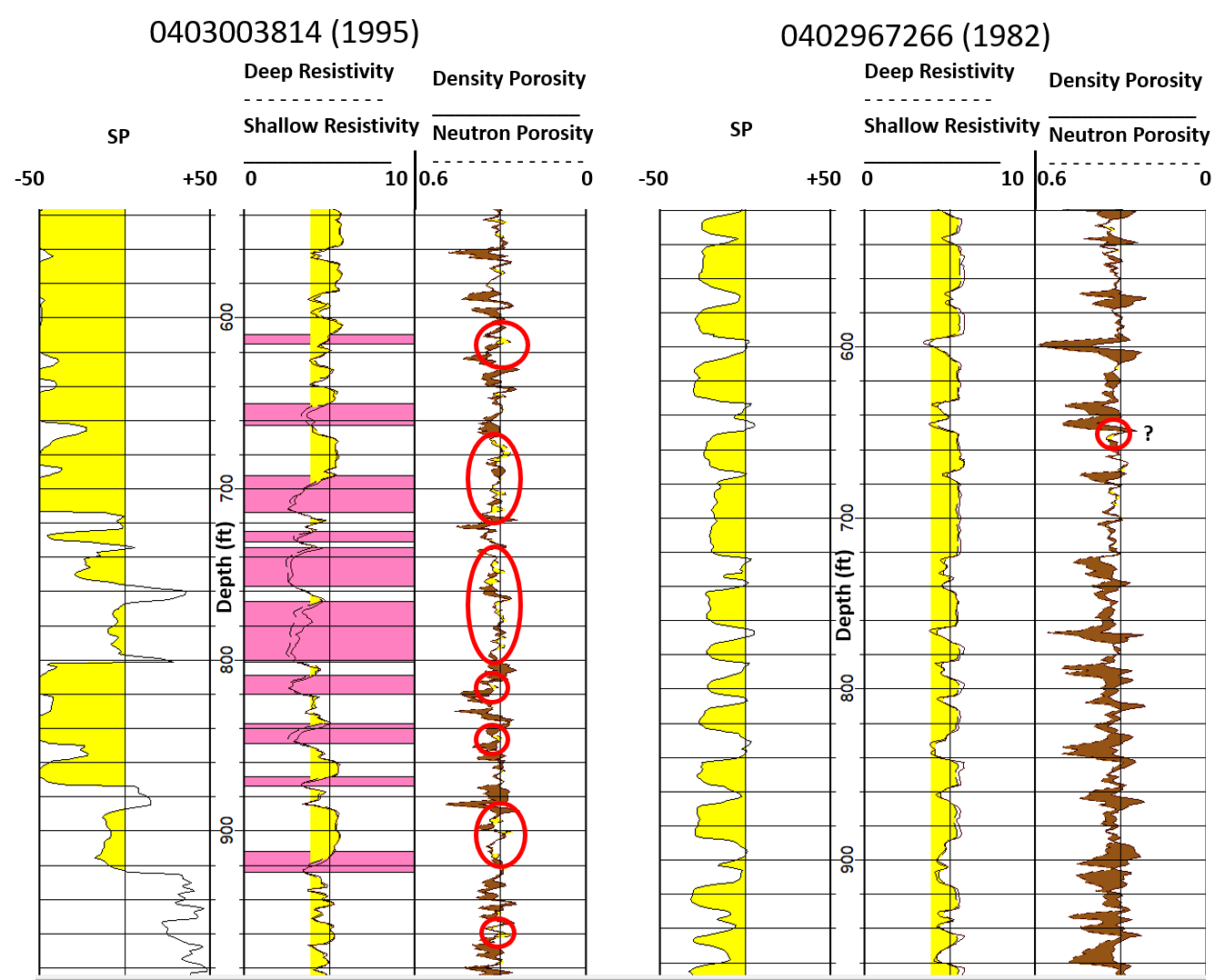

Supplement: S6 Fig — Abnormally low resistivity in the sands are highlighted in pink in the 1995 well. These intervals indicate the presence of saline produced water in the sands of the Tulare aquifer. The 1995 well also exhibits small intervals (about one meter (3 ft)) of density-neutron cross-over (shaded in yellow and highlighted by red circles) in and near the affected sands. This density-neutron behavior is common in sands affected by invasion of oilfield produced water and may indicate bacterial metabolism of traces of organic matter in the injected water and the consequent presence of methane in the sands. (TIF) [file pone.0263477.s009.tif]

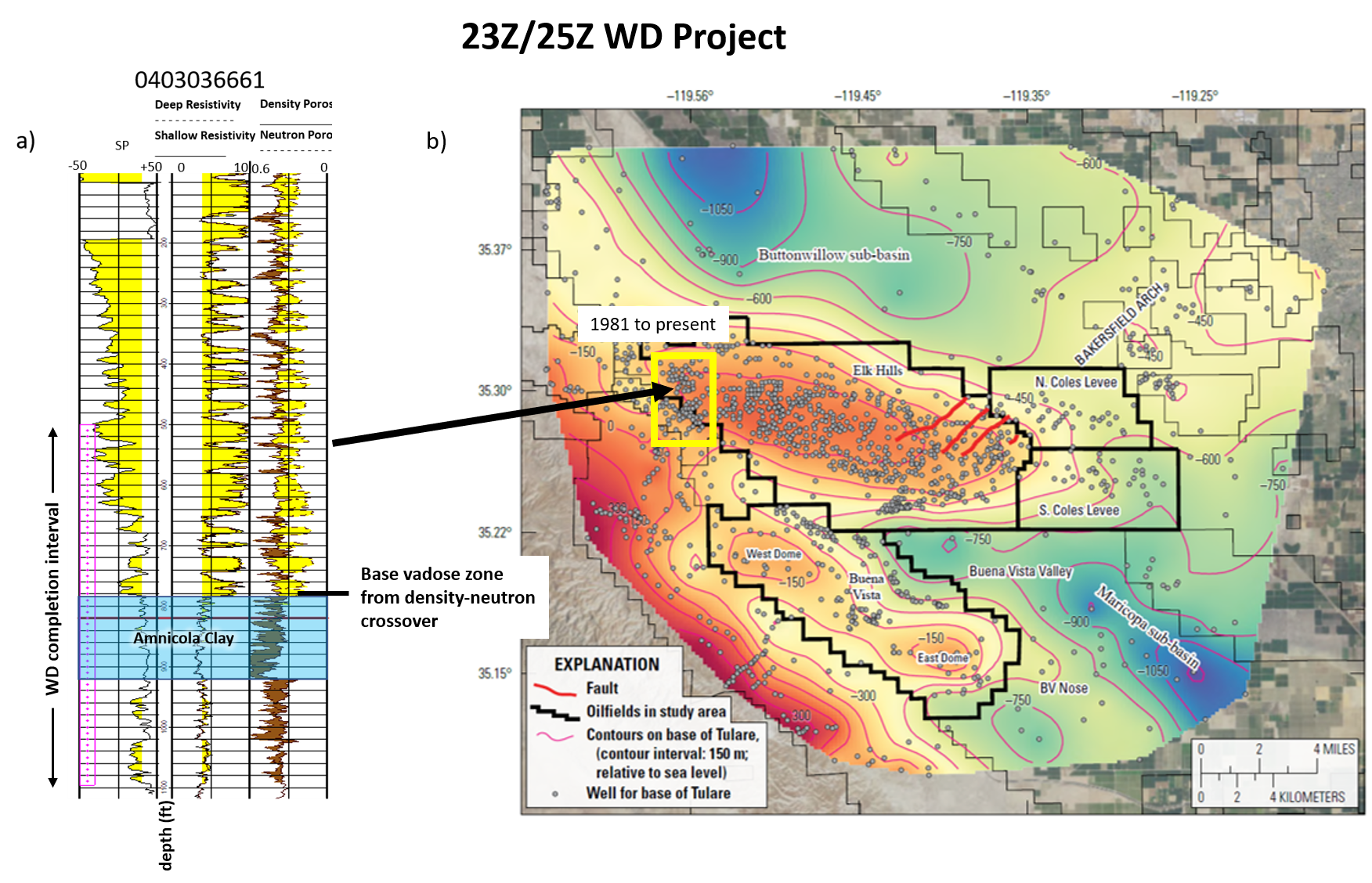

Supplement: S7 Fig — a) Type log from the 23Z/25Z water disposal (WD) project showing depth to water table from density-neutron cross-over, major clay units and completion interval. b) map showing location of the 23Z/25Z WD area in western Elk Hills (yellow box). Contours show elevation on top of the base of the Tulare Formation measured in meters with respect to mean sea level. Photographic base from https://basemap.nationalmap.gov/arcgis/rest/services/USGSImageryOnly/MapServer. (TIF) [file pone.0263477.s010.tif]

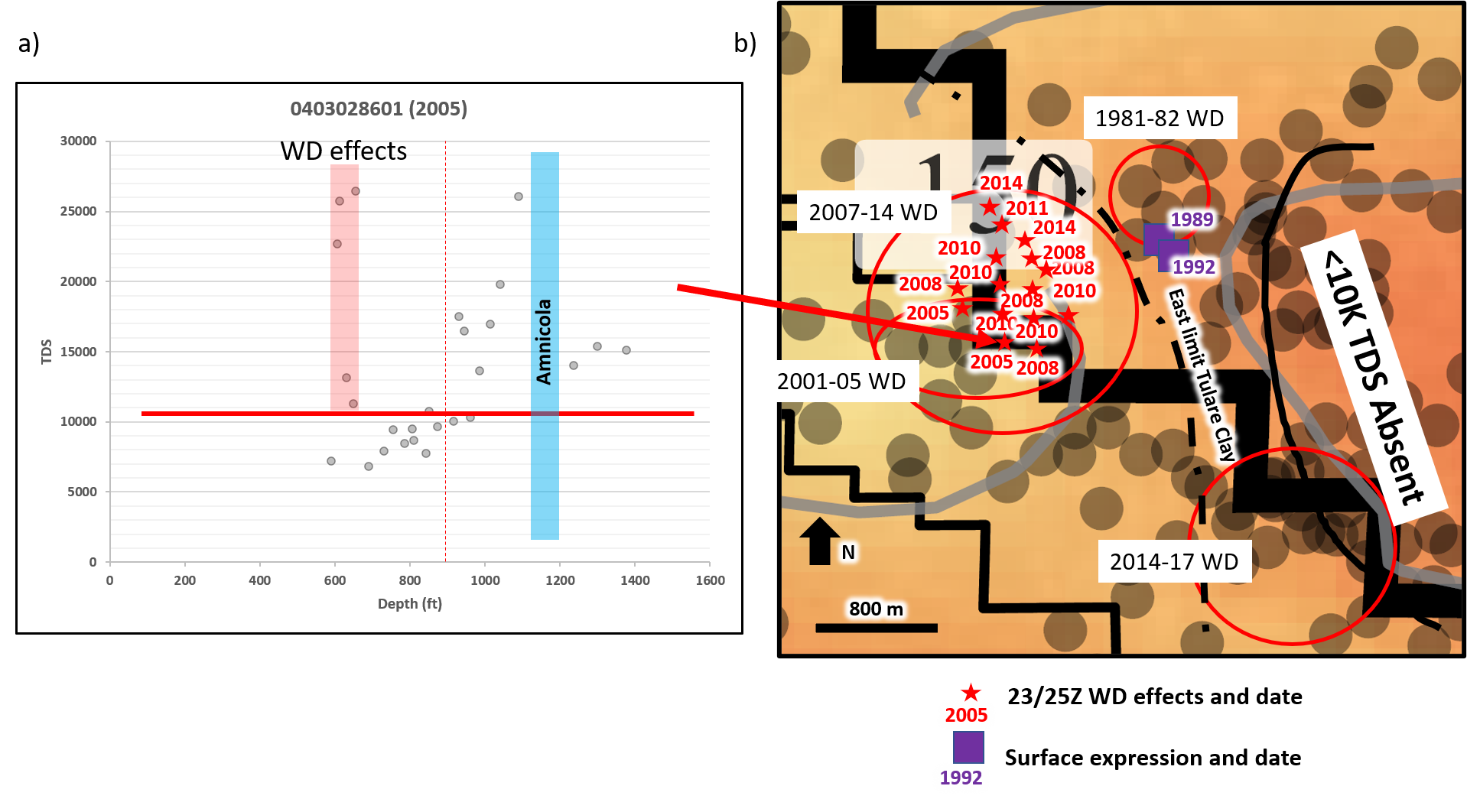

Supplement: S8 Fig — a) Example of log-calculated TDS vs depth showing effects of water disposal (WD) on salinity profile. b) Map of 23Z/25Z WD area showing the locus of produced water disposal in various years. The location and year of resistivity anomalies (red stars) and surface expressions (purple squares) caused by disposal of saline produced water into the Tulare Formation are also shown. (TIF) [file pone.0263477.s011.tif]

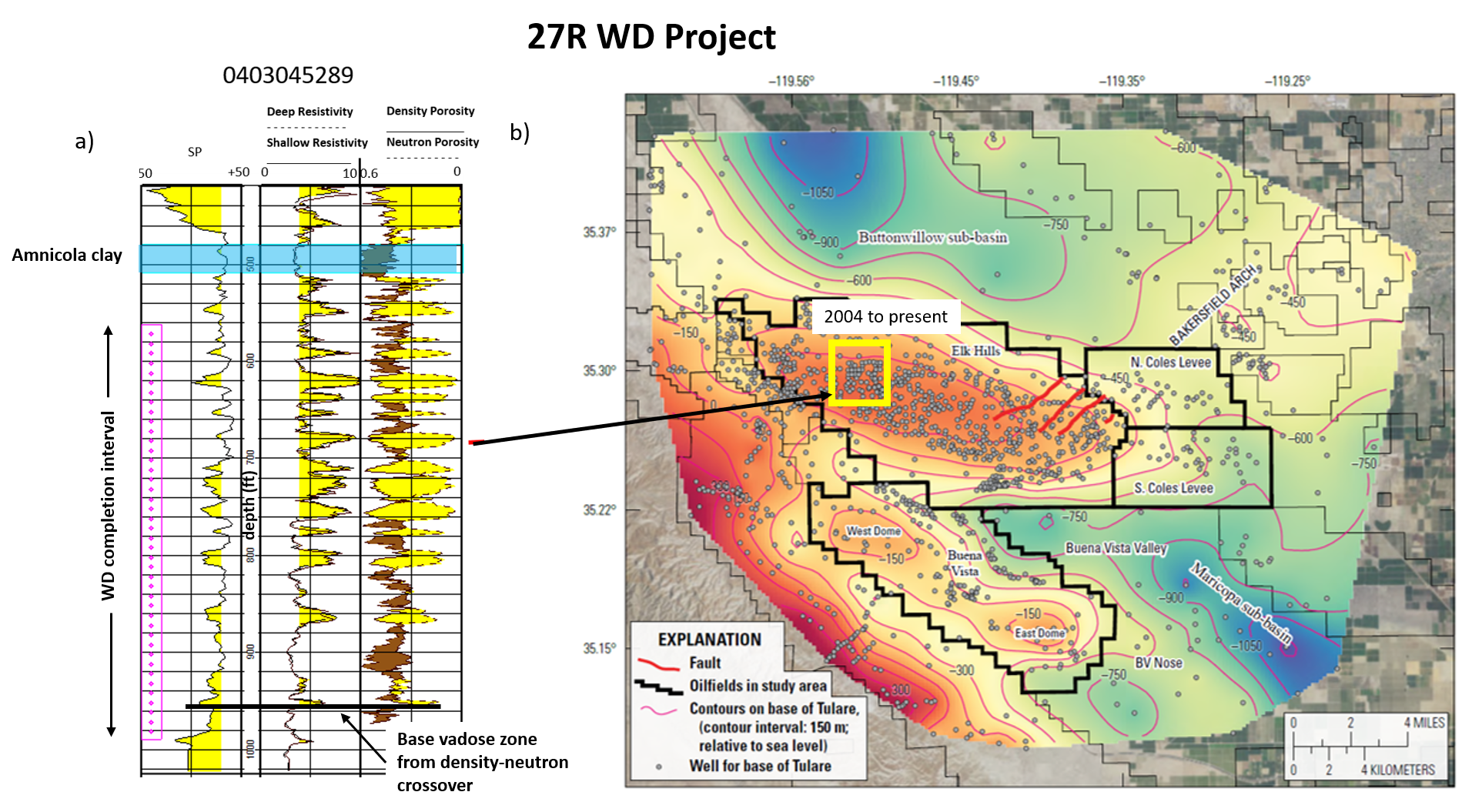

Supplement: S9 Fig — a) Type log of the Tulare Formation in the 27R WD area showing vadose zone from density-neutron logs, major clay layers and completion interval. b) Location of the 27R water disposal project area in western Elk Hills (yellow box). Contours show elevation on top of the base of the Tulare Formation measured in meters with respect to mean sea level. Photographic base from https://basemap.nationalmap.gov/arcgis/rest/services/USGSImageryOnly/MapServer. (TIF) [file pone.0263477.s012.tif]

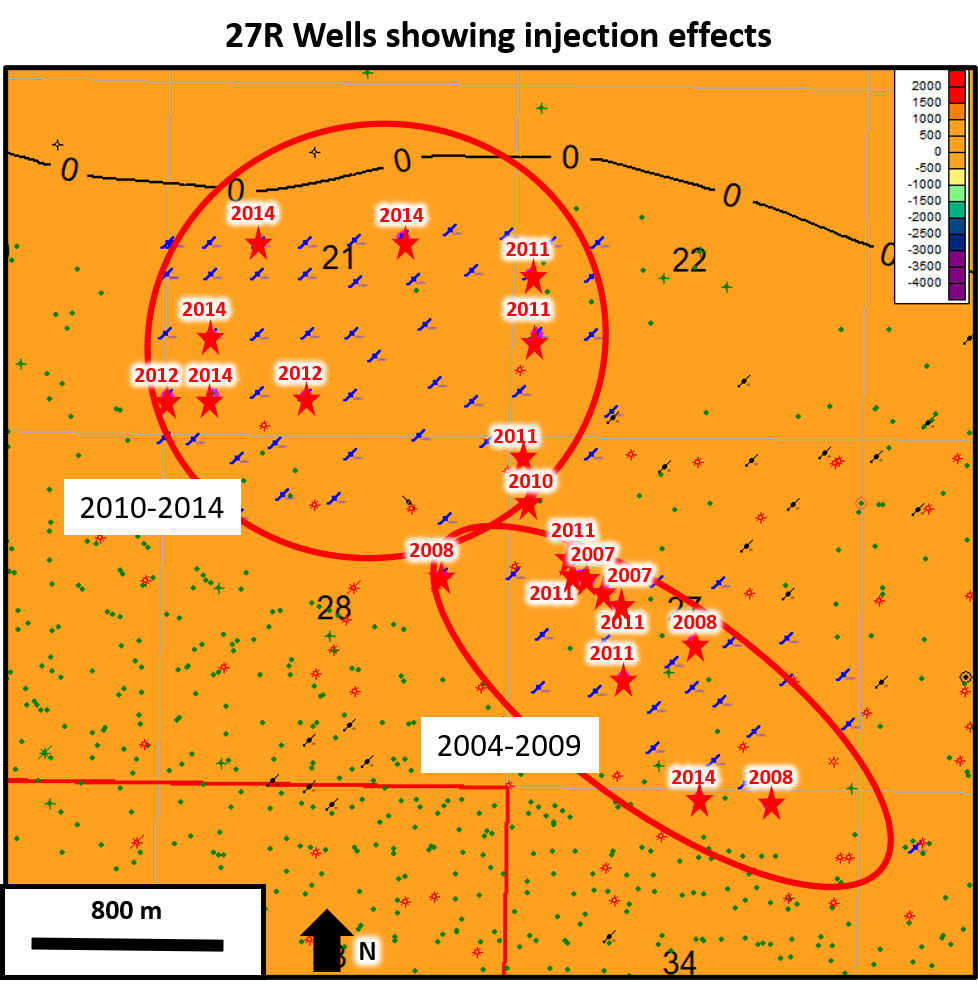

Supplement: S10 Fig — Water disposal wells are shown in blue, Enhanced oil recovery injection wells in black, gas producing wells in red and oil producing wells in green. The location and year of resistivity anomalies (red stars) caused by disposal of saline produced water into the Tulare Formation are also shown. In this case, the resistivity anomalies are noted by the fill-up of previously unsaturated sands with produced water creating saline perched aquifers above the regional water table. (TIF) [file pone.0263477.s013.tif]
