# Supplementary material for: Mapping aquifer salinity gradients and effects of oil field produced water disposal using geophysical logs: Elk Hills, Buena Vista and Coles Levee Oil Fields, San Joaquin Valley, California
Source: PLoS One. 2022 Mar 28;17(3):e0263477. doi: 10.1371/journal.pone.0263477 (PMC8959183; doi:10.1371/journal.pone.0263477)
Supplement: Attachment — Submitted filename: Response to Reviewers.docx [file pone.0263477.s015.docx]

Our responses are in red text for ease of access

Academic editor:

1. Please ensure that your manuscript meets PLOS ONE's style requirements, including those for file naming. The PLOS ONE style templates can be found at

https://journals.plos.org/plosone/s/file?id=wjVg/PLOSOne_formatting_sample_main_body.pdf and

[https://journals.plos.org/plosone/s/file?id=ba62/PLOSOne_formatting_sample_title_authors_affiliations.pdf](https://gcc02.safelinks.protection.outlook.com/?url=https%3A%2F%2Fjournals.plos.org%2Fplosone%2Fs%2Ffile%3Fid%3Dba62%2FPLOSOne_formatting_sample_title_authors_affiliations.pdf&data=04%7C01%7Cjmgillespie%40usgs.gov%7C72237c5c12cf4058c4b608d9aad36e36%7C0693b5ba4b184d7b9341f32f400a5494%7C0%7C0%7C637728647349229767%7CUnknown%7CTWFpbGZsb3d8eyJWIjoiMC4wLjAwMDAiLCJQIjoiV2luMzIiLCJBTiI6Ik1haWwiLCJXVCI6Mn0%3D%7C2000&sdata=tbfv5Z7GbrZBqznTklleieZsgdgsJ2V2Vv3tXKeUkx8%3D&reserved=0).

We think we have done this—hopefully correctly!

2. We note that the grant information you provided in the ‘Funding Information’ and ‘Financial Disclosure’ sections do not match.

When you resubmit, please ensure that you provide the correct grant numbers for the awards you received for your study in the ‘Funding Information’ section.

I don’t think there is a number associated with this funding as it is not a grant per se. The product is a deliverable for State of California agreement 19-050-250 with the U.S. Geological Survey.

3. Thank you for stating the following in the Acknowledgments Section of your manuscript:

“This work was primarily funded by the California State Water Resources Control Board Oil and Gas Regional Monitoring Program and supplemental US Geological Survey Cooperative Matching Funds.  Advice and editing from Matt Landon, Lyndsay Ball and Pete McMahon of the US Geological Survey are greatly appreciated.  Raster logs were digitized by David Shimabukuro and Theron Sower’s team of geology students from California State University Sacramento.  The appearance of the maps in the figures were greatly improved by the US Geological Survey Sacramento Publishing Service Center.”

We note that you have provided funding information that is not currently declared in your Funding Statement. However, funding information should not appear in the Acknowledgments section or other areas of your manuscript. We will only publish funding information present in the Funding Statement section of the online submission form.

Please remove any funding-related text from the manuscript and let us know how you would like to update your Funding Statement. Currently, your Funding Statement reads as follows:

“This work was primarily funded by the California State Water Resources Control Board Oil and Gas Regional Monitoring Program and supplemental US Geological Survey Cooperative Matching Funds.  The funders had no role in study design, data collection and analysis, decision to publish, or preparation of the manuscript.”

Please include your amended statements within your cover letter; we will change the online submission form on your behalf.

We have stated this in our revised cover letter and included the agreement number. Thank you for changing the online submission form for us

4. Thank you for stating the following in your Competing Interests section:

“NO”

Please complete your Competing Interests on the online submission form to state any Competing Interests. If you have no competing interests, please state ""The authors have declared that no competing interests exist."", as detailed online in our guide for authors at http://journals.plos.org/plosone/s/submit-now

 This information should be included in your cover letter; we will change the online submission form on your behalf.

We have stated this in our revised cover letter. Thank you for changing the online submission form for us

5. We note that Figures 1, 3, 4, 5, 6, 7, 9, 10, 11, 13, S3,S4, S6-S9 in your submission contain [map/satellite] images which may be copyrighted. All PLOS content is published under the Creative Commons Attribution License (CC BY 4.0), which means that the manuscript, images, and Supporting Information files will be freely available online, and any third party is permitted to access, download, copy, distribute, and use these materials in any way, even commercially, with proper attribution. For these reasons, we cannot publish previously copyrighted maps or satellite images created using proprietary data, such as Google software (Google Maps, Street View, and Earth). For more information, see our copyright guidelines: http://journals.plos.org/plosone/s/licenses-and-copyright.

 We require you to either (1) present written permission from the copyright holder to publish these figures specifically under the CC BY 4.0 license, or (2) remove the figures from your submission:

 a. You may seek permission from the original copyright holder of Figure 1, 3, 4, 5, 6, 7, 9, 10, 11, 13, S3,S4, S6-S9 to publish the content specifically under the CC BY 4.0 license.

 We recommend that you contact the original copyright holder with the Content Permission Form (http://journals.plos.org/plosone/s/file?id=7c09/content-permission-form.pdf) and the following text:

“I request permission for the open-access journal PLOS ONE to publish XXX under the Creative Commons Attribution License (CCAL) CC BY 4.0 (http://creativecommons.org/licenses/by/4.0/). Please be aware that this license allows unrestricted use and distribution, even commercially, by third parties. Please reply and provide explicit written permission to publish XXX under a CC BY license and complete the attached form.”

 Please upload the completed Content Permission Form or other proof of granted permissions as an ""Other"" file with your submission.

 In the figure caption of the copyrighted figure, please include the following text: “Reprinted from [ref] under a CC BY license, with permission from [name of publisher], original copyright [original copyright year].”

 b. If you are unable to obtain permission from the original copyright holder to publish these figures under the CC BY 4.0 license or if the copyright holder’s requirements are incompatible with the CC BY 4.0 license, please either i) remove the figure or ii) supply a replacement figure that complies with the CC BY 4.0 license. Please check copyright information on all replacement figures and update the figure caption with source information. If applicable, please specify in the figure caption text when a figure is similar but not identical to the original image and is therefore for illustrative purposes only.

The following resources for replacing copyrighted map figures may be helpful:

USGS National Map Viewer (public domain): http://viewer.nationalmap.gov/viewer/

The Gateway to Astronaut Photography of Earth (public domain): http://eol.jsc.nasa.gov/sseop/clickmap/

Maps at the CIA (public domain): https://www.cia.gov/library/publications/the-world-factbook/index.html and https://www.cia.gov/library/publications/cia-maps-publications/index.html

NASA Earth Observatory (public domain): http://earthobservatory.nasa.gov/

Landsat: http://landsat.visibleearth.nasa.gov/

USGS EROS (Earth Resources Observatory and Science (EROS) Center) (public domain): http://eros.usgs.gov/#

Natural Earth (public domain): [http://www.naturalearthdata.com/](https://gcc02.safelinks.protection.outlook.com/?url=http%3A%2F%2Fwww.naturalearthdata.com%2F&data=04%7C01%7Cjmgillespie%40usgs.gov%7C72237c5c12cf4058c4b608d9aad36e36%7C0693b5ba4b184d7b9341f32f400a5494%7C0%7C0%7C637728647349239722%7CUnknown%7CTWFpbGZsb3d8eyJWIjoiMC4wLjAwMDAiLCJQIjoiV2luMzIiLCJBTiI6Ik1haWwiLCJXVCI6Mn0%3D%7C2000&sdata=B80dbDAmoAF2QcfrdqRxInVsEwNvBpSw1mqvsqeX8x0%3D&reserved=0)

The basemap for figures 3, 5, 6, 9, 10, 11, 13, S4, S7 and S9 are from the USGS National Map (https://basemap.nationalmap.gov/arcgis/rest/services/USGSImageryOnly/MapServer/). The basemaps for Figures 1 and 13 were created in ArcMap by the authors. The basemap in figure 4 was created in IHS Petra software by the authors. The basemap for figures 7 and S5 are from arcgisonline (<http://server.arcgisonline.com/arcgis/rest/services/Elevation/World_Hillshade/MapServer>). The basemaps from the USGS National Map and arcgisonline are in the public domain.

Reviewer #1: The authors reported an excellent research on the investigation of Mapping aquifer salinity gradients and effects of oil field produced water disposal using geophysical logs. The manuscript is within the journal scope and has the potential to be accepted. However, there are several comments to be resolved before the manuscript publication.

1- The abstract is too lengthy, it shall be revised to present only the essential information and in one single paragraph.

We are unclear why the reviewer notes that the abstract is too long as he/she did not note what non-essential information should be removed. PLoS One guidelines for submission note the following:

“The Abstract should:

- Describe the main objective(s) of the study (In our abstract this is found in Lines 25-28)
- Explain how the study was done, including any model organisms used, without methodological detail (Lines 29-31)
- Summarize the most important results and their significance (Lines 32-40—effects of oil and gas production on protected aquifers)
- Not exceed 300 words (Our submitted abstract was 264 words and the current abstract (after revisions) is 283—below the 300-word limit)”

The first 3 lines of the abstract (23-25), note why the study was undertaken. Perhaps the reviewer wishes us to remove these 3 lines? But then we will not address the significance of the work or why it was done.

We addressed this comment by combining the paragraphs in the abstract into a single paragraph

2- All abbreviations shall be defined once you have mentioned at first. Example TDS, etc. Yes. Thank you. We have double checked the paper for these and made corrections.

3- Modeling flowchart should be included in the method section for better understanding by the readers.

We have included flow charts for the water surface elevation modeling and the groundwater salinity calculations in the supplement and referred to these in the Methods section

4- As per the overlapping of the manuscript: 8% was observed related to the following document
https://archives.datapages.com/data/deg/2019/EG032019/eg18009/eg18009.html
Authors are responsible to reduce the plagiarism percentage for their ethical right.

Using the software at <https://desklib.com/writing/compare/>, the authors confirmed the 8% overlap with [Gillespie and others (2019)](https://pubs.geoscienceworld.org/eg/article/26/3/73/573555/Groundwater-salinity-and-the-effects-of-produced) as noted by the reviewer. The main areas of overlap were in the sections describing the geologic background of the San Joaquin Valley and the description of the method used to determine salinity using geophysical logs. Some overlap involved direct quotes from cited publications. In response to this comment, the overlapping sections were reworded, and additional citations were added, which resulted in reducing the overlap to 1%.

5- A new section presenting the practical aspect of the attained results shall be reported in the manuscript.

We added several paragraphs to the beginning of the Discussions and Conclusions section noting how our research relates to issues in the San Joaquin Valley and, more generally, to help determine salinity in brackish water aquifers where samples are often difficult to obtain.

6- English editing is recommended for the article.

We have done this

Reviewer #2: The manuscript “Mapping aquifer salinity gradients and effects of oil field produced water disposal using geophysical logs: Elk Hills, Buena Vista and Coles Levee Oil Fields, San Joaquin Valley, California”; is technically sound, and data support the conclusions. The conclusions consistent with the evidence and arguments presented. Although it is relevant, interesting and well written, but it is not easy to read. Tables and figures aid understanding, but I think, author used huge number of figures, and some of them are superfluous. However, However, many notes listed below have emerged that need to be addressed, and without they would not be accepted for publication. Consequently, I recommend a major revision.

1. In order to complete the elements of the introduction, the previous studies must be mentioned, so I find that some sentences in the introduction need to be reformulated to show the problem of the research and its importance clearly, and what is the new addition (novelty) to the field of knowledge after listing the relevant conclusions of previous researches. At the end of this report, I have put a number of useful links that can be quoted to support some ideas and scientific facts.

Reference to the articles suggested by the reviewer were not added because the authors did not believe they were applicable to the focus of this manuscript study, which covers relatively young (late Cenozoic) clastic reservoirs/aquifers in California, US, rather than late Mesozoic carbonate reservoirs in Iraq. In addition, the method of isotopic analysis to determine the origin of the brines in the suggested reference articles is very different from the analysis of saline and brackish waters in this study. The authors added four new references to the Introduction section that pertain more directly to this study.

2. Line 57: Please delete “milligrams per liter”, It is enough to be mentioned by “mg/l”.

PLOS guidelines and Reviewer #1 note that the use of each abbreviation should be defined when it is first mentioned in the article. Therefore, we think this needs to remain as written as this is its first occurrence in the paper. And the L should be capitalized as per standards.

3. Line 77: What do you mean by “native waters”, do it mean fresh water, if yes, please use the last term.

By native waters we do not mean fresh waters but the water originally in the aquifer prior to anthropogenic disruption such as injection of produced waters from oilfields. These native formation waters are not fresh in most cases but more commonly brackish (TDS between 1000 and 10,000 mg//L TDS; <https://water.usgs.gov/ogw/gwrp/brackishgw/brackish.html>; accessed 10/6/2021). Therefore, it would be erroneous to refer to them as fresh. We removed the term “native” from the sentence and replaced it with “brackish” to avoid this confusion.

4. Line 83-84: The Elk Hills Oil Field (formerly Naval Petroleum Reserve No.1) has produced over one billion barrels (160,000,000 m3) of oil; Please fix the period of time (year) during which that huge amount of oil was produced.

That is a good suggestion and we added text to the revised manuscript indicating this is cumulative production since the field was discovered in 1911. Thank you.

5. Fig. 1: The red line represented the San Andreas fault zone appears to be extended somewhat outside the map boundary. Please correct.

Good point. Thank you! This has been corrected.

6. Fig. 2: There are four rectangles that appear as blanks of white color! What do they represent? Could you add the thickness of the Tulare aquifer, please? There are three beds of clay. Is aquifer a multi-confined aquifer. What is a water-bearing lithology?.

The white boxes are meant to represent the sandy water-bearing zones of the aquifer that lie between the regional clay beds. We have added lithologic symbols to the boxes to make them more identifiable as sand lithology. The thickness of the Tulare aquifer is shown in Figure 5. Line 255 notes that the Tulare aquifer is characterized as leaky (i.e. leaky confined). We have moved that statement to a position earlier in the aquifer description.

7. Line 108: The abbreviation “SJV forms” comes suddenly, please define it at the first appearance.

This was defined in Line 81 of the manuscript so we don’t believe it needs to be re-defined here as this is not the first occurrence of the acronym in the paper. We thank the reviewer for making this point because it lead us to notice that acronym for San Joaquin Valley was not used in two locations (L129, L145) where it should have been used in the Geologic Setting section. That mistake has been corrected in the revised manuscript.

8. Fig.3: This fig is of low resolution.

Thank you. We have submitted a clearer version of the figure.

9. Line 180: Please, it is enough to write 23 cm) (5 to 9 in, and not necessary to write in detail as 23 centimeters (cm) (5 to 9 inches (in)).

As per reviewer #1 and PLOS guidelines, all abbreviations should be defined at first use (see comment #2 from reviewer #1)

10. Line 182: The same comment above for “centimeters per year (cm/yr) (6 inches per year (in/yr)).

As per reviewer #1 and PLOS guidelines, all abbreviations should be defined at first use (see comment #2 from reviewer #1)

11. Lines 212-213: “The contact between the Tulare and the overlying alluvium was placed at a depth of 144 m (473 ft) in well 02938955 in the Buena Vista Valley”; where is the location on map of this well?.

Good point. We have noted the location of this well in Figure 3.

12. For the better presentation of the hydrological system, you need a 3D model for the aquifer.

Adding a 3D aquifer model is beyond the scope of this study and therefore no change was made in response to this comment. Moreover, regional 3D models of the Tulare aquifer are already available. We have added references to these published works at the beginning of the description of the Tulare aquifer.

13. Caption of Table 1 should be placed above (not bellow) table.

Thank you. This has been corrected.

14. Method section: It has been written very briefly; I would prefer to rewrite it in details.

The request to rewrite the Methods section in more detail is somewhat vague but, given that Reviewer #1 requested flow charts be included, inclusion of these charts provides additional detail regarding the methods.

15. The date of water sampling is very important to be mentioned, particularly for the water quality.

Thank you. This information is included in the accompanying data release referred to in the article Stephens MJ, Gillespie JM, Chang W. and Warden J. Geophysical, geological, and geochemical data for aquifer salinity mapping in the Elk Hills Oil Field, Kern County, California: U.S. Geological Survey data release, in prep so will be available to the readers at the time of publication in PLOS One.

16. Line 347-348; the mention to the figures should be sequentially, here in the statement “Geophysical well log correlations were used to map the elevation of the base of the Tulare Formation (Fig 3) and its thickness (Fig 5)”, author has mentioned FIG 5 Before mentioning to Fig 4.

Figure 4 is referred to in Line 310 so it precedes the reference to figure 5 in Line 347

17. Line 364: the site cannot be reached.

We were able to access the site on 10/6/2021 by clicking on the link in the text. The page should appear as follows:

18. One of the weaknesses of the methods is the lack of regularly distributed wells. The other thing is determining the net clay thickness without using core data.

We agree that, in an ideal world, all wells would be regularly spaced throughout an area and complete sediment cores would be available across the aquifer at all sites. However, in most oil-producing areas, wells are concentrated within the productive limits of the field and wells are more sparsely distributed outside the producing areas where commercial resources do not exist. We regret that we do not have uniformly distributed well data but we feel that, by including exploration wells and water production wells outside the field limits, we have compensated for this to the best of our ability. As for core data, because the Tulare Formation does not produce oil in the study area, it is not extensively cored. Only two wells were cored in the Tulare Formation and they are not uniformly distributed within the study area and do not cover the entire formation. Therefore, we used the separation of the density-neutron geophysical log curves to determine clay content because these logs are relatively abundant and occur throughout the study area. This technique is commonly used in the oil industry to determine net clay values within oil reservoirs (<https://www.spec2000.net/11-vshnd.htm>; accessed 11/3/2021), (<https://geoloil.com/VshFromNeutrDens.php>, accessed 11/3/2021).

19. Why was the figure numbering system changed to S1-S9 after Fig 13?

The figures preceded by S are referred to in the supplemental section of the paper and are not part of the main body of the paper. PLoS One submission guidelines state that: “You may use almost any description as the item name of your supporting information as long as it contains an "S" and number. For example, “S1 Appendix” and “S2 Appendix,” “S1 Table” and “S2 Table,” and so forth…. Match the names of your supporting information files with the supporting information captions within your manuscript. For example, a PDF file for “S2 Fig.” must be named “S2_fig.pdf.” To address this issue, the revised manuscript now collates the supplementary figure captions at the end of the manuscript in the supplementary material section. We apologize for the confusion.

20. Line 702: Caption of Table 2 must be above the table not bellow.

Thank you. We have corrected this.

21. Line 754-755: “This suggests that the saline injected water and brackish formation waters are segregated by density and mixing has not had time to occur in some sands”. It is very difficult to believe this suggestion for the simple reason that salinity is represented by positive and negative ions and these ions can move quickly in the solution, which quickly becomes homogeneous with time and after a short period

Papers noting a boundary between overlying fresh water and underlying saline water in porous media were first published in the 19^th^ century by Du Commun (1828), Baydon-Ghyben (1888-1889) and Herzberg (1901). In fact, Fetter (2001) in his classic textbook “Applied Hydrogeology” notes that “if fresh ground water is adjacent to saline ground water, the difference in density between the two fluids becomes very important.” The presence of a mixing zone of various thickness between freshwater and underlying saline water forms the basis for efforts to inhibit salt-water encroachment and to develop aquifer storage and recovery (ASR) projects in coastal aquifers. In aquifer design scenarios for freshwater ASR projects in island and coastal areas, van Ginkel (2015) notes that “An initial vertical interface between two fluids with different densities, as it develops after a vertical ASR-well starts injecting, will rotate” and “as time passes, the two fluids stratify with the denser fluid spreading along the bottom and the lighter fluid accumulating on the top of the aquifer.” More pertinent to our observations is the research of Shincariol and Schwartz (1990) in which saline water of varying salinities (1000, 2000, 10,000 and 100,000 mg/L NaCl) was injected into an experimental tank filled with layered porous media containing fresh water—a scenario similar to the injection of saline produced water into brackish water aquifers in our study. They noted that “The denser, more saline plumes sank to the bottom of the tank after 13 hours” and “dense plumes mounded above the boundary with a low conductivity unit and behaved in a manner not unlike that of a dense, non-aqueous phase liquid.” We note similar behavior by injected saltwater into a brackish water aquifer in our study—the injected saline water appears to form a plume that flows along the base of the sand bodies until they are filled. We have referred to the experiments of Shincariol and Schwartz (1990) and have noted their results in the Results section of our paper to reinforce the validity of our statements about the behavior of saline produced water within a brackish aquifer. Shincariol and Scwartz also noted that “in dipping layers, the density forces provide a component of flow down the topographic gradient of the interface” (ie. down the dip of the layers). This is similar to our observations on the southward (downdip) movement of saline injected fluids on the south flank of the Elk Hills anticline so we also included this statement in our Discussions and Conclusions section.

22. Many useful papers can be cited as suitable, Introductas:
- https://link.springer.com/article/10.1007/s11053-021-09923-4
- https://www.sciencedirect.com/science/article/abs/pii/S0264817220304207
- https://link.springer.com/article/10.1007/s13201-019-0944-6
- https://link.springer.com/chapter/10.1007/978-3-030-01572-5_12
- https://jprs.gov.iq/index.php/jprs/article/view/273
- https://link.springer.com/article/10.1007/s12517-018-3908-5
- <https://link.springer.com/article/10.1007/s40808-018-0510-5>

The authors thank the reviewer for introducing us to his interesting studies on the origin of brines in Mesozoic carbonate reservoirs in Iraq. However, as noted earlier, the methods and the focus of these studies are very different from our studies on brackish water in late Cenozoic clastic aquifers in California as we use calculated salinity (as NaCl) from geophysical logs verified by TDS values from standard lab analyses to determine salinity and not isotopes. We have added four references to the Introduction that are more pertinent to this study in order to address this comment.

While revising your submission, please upload your figure files to the Preflight Analysis and Conversion Engine (PACE) digital diagnostic tool, [https://pacev2.apexcovantage.com/](https://gcc02.safelinks.protection.outlook.com/?url=https%3A%2F%2Fpacev2.apexcovantage.com%2F&data=04%7C01%7Cjmgillespie%40usgs.gov%7Cab6db299d4f8455d99b608d987a1f8ab%7C0693b5ba4b184d7b9341f32f400a5494%7C0%7C0%7C637689952450614058%7CUnknown%7CTWFpbGZsb3d8eyJWIjoiMC4wLjAwMDAiLCJQIjoiV2luMzIiLCJBTiI6Ik1haWwiLCJXVCI6Mn0%3D%7C0&sdata=FakZeU5f%2FiT3kJjbZCjrJNW2HNia8rxj1t1vUbo8Zl0%3D&reserved=0). PACE helps ensure that figures meet PLOS requirements. To use PACE, you must first register as a user. Registration is free. Then, login and navigate to the UPLOAD tab, where you will find detailed instructions on how to use the tool. If you encounter any issues or have any questions when using PACE, please email PLOS at [figures@plos.org](mailto:figures@plos.org). Please note that Supporting Information files do not need this step.

The figures have been uploaded to PACE
