# Supplementary material for: Mapping aquifer salinity gradients and effects of oil field produced water disposal using geophysical logs: Elk Hills, Buena Vista and Coles Levee Oil Fields, San Joaquin Valley, California
Source: PLoS One. 2022 Mar 28;17(3):e0263477. doi: 10.1371/journal.pone.0263477 (PMC8959183; doi:10.1371/journal.pone.0263477)
Supplement: Attachment — Submitted filename: Reviewer comments.docx [file pone.0263477.s014.docx]

**Reviewer comments**

The manuscript “Mapping aquifer salinity gradients and effects of oil field produced water disposal using geophysical logs: Elk Hills, Buena Vista and Coles Levee Oil Fields, San Joaquin Valley, California”; is technically sound, and data support the conclusions. The conclusions consistent with the evidence and arguments presented. Although it is relevant, interesting and well written, but it is not easy to read. Tables and figures aid understanding, but I think, author used huge number of figures, and some of them are superfluous. However, However, many notes listed below have emerged that need to be addressed, and without they would not be accepted for publication. Consequently, I recommend a major revision.

1. In order to complete the elements of the introduction, the previous studies must be mentioned, so I find that some sentences in the introduction need to be reformulated to show the problem of the research and its importance clearly, and what is the new addition (novelty) to the field of knowledge after listing the relevant conclusions of previous researches. At the end of this report, I have put a number of useful links that can be quoted to support some ideas and scientific facts.
2. Line 57: Please delete “milligrams per liter”, It is enough to be mentioned by “mg/l”.
3. Line 77: What do you mean by “native waters”, do it mean fresh water, if yes, please use the last term.
4. Line 83-84: The Elk Hills Oil Field (formerly Naval Petroleum Reserve No.1) has produced over one billion barrels (160,000,000 m3) of oil; Please fix the period of time (year) during which that huge amount of oil was produced.
5. Fig. 1: The red line represented the San Andreas fault zone appears to be extended somewhat outside the map boundary. Please correct.
6. Fig. 2: There are four rectangles that appear as blanks of white color! What do they represent? Could you add the thickness of the Tulare aquifer, please? There are three beds of clay. Is aquifer a multi-confined aquifer. What is a water-bearing lithology?.
7. Line 108: The abbreviation “SJV forms” comes suddenly, please define it at the first appearance.
8. Fig.3: This fig is of low resolution.
9. Line 180: Please, it is enough to write 23 cm) (5 to 9 in, and not necessary to write in detail as 23 centimeters (cm) (5 to 9 inches (in)).
10. Line 182: The same comment above for “centimeters per year (cm/yr) (6 inches per year (in/yr)).
11. Lines 212-213: “The contact between the Tulare and the overlying alluvium was placed at a depth of 144 m (473 ft) in well 02938955 in the Buena Vista Valley”; where is the location on map of this well?.
12. For the better presentation of the hydrological system, you need a 3D model for the aquifer.
13. Caption of Table 1 should be placed above (not bellow) table.
14. Method section: It has been written very briefly; I would prefer to rewrite it in details.
15. The date of water sampling is very important to be mentioned, particularly for the water quality.
16. Line 347-348; the mention to the figures should be sequentially, here in the statement “Geophysical well log correlations were used to map the elevation of the base of the Tulare Formation (Fig 3) and its thickness (Fig 5)”, author has mentioned FIG 5 Before mentioning to Fig 4.
17. Line 364: the site cannot be reached.
18. One of the weaknesses of the methods is the lack of regularly distributed wells. The other thing is determining the net clay thickness without using core data.
19. Why was the figure numbering system changed to S1-S9 after Fig 13?
20. Line 702: Caption of Table 2 must be above the table not bellow.
21. Line 754-755: “This suggests that the saline injected water and brackish formation waters are segregated by density and mixing has not had time to occur in some sands”. It is very difficult to believe this suggestion for the simple reason that salinity is represented by positive and negative ions and these ions can move quickly in the solution, which quickly becomes homogeneous with time and after a short period
22. Many useful papers can be cited as suitable, Introductas:

- <https://link.springer.com/article/10.1007/s11053-021-09923-4>
- <https://www.sciencedirect.com/science/article/abs/pii/S0264817220304207>
- <https://link.springer.com/article/10.1007/s13201-019-0944-6>
- <https://link.springer.com/chapter/10.1007/978-3-030-01572-5_12>
- <https://jprs.gov.iq/index.php/jprs/article/view/273>
- <https://link.springer.com/article/10.1007/s12517-018-3908-5>
- <https://link.springer.com/article/10.1007/s40808-018-0510-5>
